# The Application of Chemistry to Dentistry

**Published:** 1862-01

**Authors:** Charles M. Wetherill


### DENTAL REGISTER OF THE WEST.

VOL. XVI.] JANUARY, 1862. [No. 1.

### Original Essays and Communications.

### THE APPLICATION OF CHEMISTRY TO DENTISTRY.

BY CHARLES M. WETHERILL, PH., D., M. D.

(Read before the Indiana State Dental Association, January 15., 1862.)

By the ancient Jewish law "an eye for an eye and a tooth for a tooth" must be given. In this law of retaliation the loss of the tooth stands next in importance to that loss, acknowledged by all to be the greatest, by which we are deprived of the light of nature and the countenance of our friends.

Whoever then in our day strives to make good such bodily losses, deserves well of his fellow beings, like him who causes two blades of grass to grow, where before there grew but one.

Chemistry, in extending her influence over the natural sciences, has not denied her aid to dentistry ; but has been successfully invoked. Dentistry in our Country is a *live* art; she speedily adopts applications from the various sciences, and it is doubtless owing to this practical spirit of her votaries, that we outshine the rest of the civilized world in all that concerns the teeth. Indeed, dentistry has attained her present importance in the United States through a purely practical endeavor, that of supplying artificial teeth. It is this invention that has taken the art of tooth drawing from the hands of the surgeon, and has increased the number of practicing dentists from thirty, in the year 1820, to over five thousand at the present time. It has been estimated that, in twenty years, the number of artificial teeth made here annually, has increased from two hundred and fifty thousand to five millions. A large number of them are exported. We supersede the French, who were the pioneers in this branch of industry, and the capital employed is estimated at upwards of five hundred thousand dollars.

One firm alone uses three hundred ounces of platinum monthly for the pins of porcelain teeth, of which it manufactures one hundred and eighty thousand per month. The same firm sells $109,200 worth of gold foil annually, and it is estimated that the dentists of our country employ two millions and a half dollars worth of gold each year.*

I have been invited to read an article upon some of the applications of chemistry to dentistry, before the members of this Convention. If my remarks should prove too hacknied, I claim the indulgence of the Society, from the shortness of the notice, as well as from the fact that my reading does not bring me in contact with the numerous and well appointed Journals of Dental Science.

There are two general propositions which embrace, perhaps, all the aid which dentistry demands from chemistry in the present state of the science; these are:

I. The supplying a cheaper and, if possible, a better material for the plates etc., of porcelain teeth.

II. Improvements upon the present modes of the arrest of the tooth's decay, whether by furnishing a cheaper and better material for filling the cavities; or (either alone or hand in hand with physiology) by discovering the cause and cure of the decay.

1. In considering the first of these propositions it may be remarked that the porcelain teeth themselves, in appearance, hardness, indestructibility, cheapness, &c., supply every desid-

---

*Ann. Sc. Discovery, 1861, p 91.

cratum; but that great improvements are needed in the material of the machinery which renders artificial teeth available for the purposes for which they are intended.

What is desirable in this respect is plain even to the tyro. There is needed a cheap material of low specific gravity, and with the necessary stability to endure the work to which the plates are subjected. The material must also be such that it is not corroded or decomposed to any great extent by the fluids with which it comes in contact; if trifling oxidation does result, it is absolutely requisite that no poisonous compounds be formed. The material must furthermore be of a compact character, offering no pores for the retention and subsequent decomposition of organic matter. But it is one thing to be aware of a want, and a quite different thing to be able to supply it. It is no wonder, then, that while the porcelain teeth soon reached perfection, the material of their setting generally remains what it originally was; viz: gold for the rich, and a much baser metal for the million. To a chemist, the prospective improvement in the material for setting artificial teeth appears to be confined to two substances: 1st. Hard vulcanized rubber, further improved and adapted to the purpose; and 2d, the so called new metals and their alloys which are attracting so much attention in the world of applied chemistry.

As the former article is already in the hands of dentists who are experiencing its capabilities, and as the nature of the substance itself has become pretty well known to the public, I will confine myself to a brief statement of the nature and properties of the new metals.

These metals, so new to the general public, are, notwithstanding, of respectable antiquity for chemical products.

In 1807, Sir Humphrey Davy communicated to the Royal Society his discovery, by the aid of the galvanic current, of the metals, potassium and sodium, contained respectively in the alkalies potassa and soda, which are the oxides of the metals. Not long afterward the metals were obtained, by chemical means, by strongly heating the alkaline carbonates, mingled intimately with carbon. A great impulse was thereby given to the study of metallic chemistry. So strong is the affinity of the alkaline metals for oxygen, that, when they are heated with any of the other metallic oxides, they deprive them of oxygen, reducing them to the metallic state, and returning themselves to the condition of alkalies. Thus lime, baryta, strontia, magnesia, alumina &c., were soon proved to be metallic oxides, and their metals were isolated in small quantities. Even ammonia did not escape these searches after truth; its alkaline character awakened the suspicion that it was the oxide of, a perhaps gaseous metal, ammonium, and although this metal has not been isolated, its amalgam, which strongly resembles the amalgams of other metals, has been prepared. Owing to theexpense of obtaining them, the metals alluded to were not prepared in sufficient quantities to study fully their properties. For the same reason they were not introduced into the arts until a few years ago, when the French chemist Deville took up the subject, and after having discovered means of obtaining sodium sufficiently cheap, succeeded in placing these metals, and especially aluminium, to which his attention was chiefly directed, within the scope of the arts. With respect to the price at which aluminium and the kindred metals may be furnished, the subject remains, in one respect, where Deville found it. This chemist introduced the metal by cheapening sodium, and whatever renders sodium still cheaper, will make aluminium still more available. The ores, so to say, of sodium and aluminium are found everywhere; they are so cheap as to be of but little value. The process of their reduction has only to be improved, and we may confidently expect that it will be done, to make these metals more common than tin. There was a day in the history of man when iron was dearer than aluminium is now.

Without desiring to detract from the applications of the other similar metals to dental purposes, I will confine my remarks to aluminium, since at present it alone is accessible, to dentists. The properties of the other metals are rapidly becoming better known, and since such properties as would render them useful in the arts would make them no less useful in dentistry, they will become available to dentists upon the discovery of such properties.

In the present state of the aluminium manufacture it is necessary to first make sodium. This metal is obtained by the action of a strong heat upon an intimate and finely pulverized and dry mixture, of thirty parts crystallized carbonate of soda, thirteen coal, and five chalk, contained in clay or metal vessels. The sodium being volatile, distils over, and is condensed, with precautions taken to prevent too free a contact with air while it is yet warm.

Sodium and potassium are well known to all who have attended elementary chemical lectures. They are soft like wax; of the lowest specific gravityof all the metals (potassium being the lowest), they float upon water, which they decompose, evolving its hydrogen, and combining with its oxygen; heat is thereby generated, which, in the case of potassium, ignites the hydrogen, which burns with a flame colored violet by the fumes of the metal. Sodium, being better adapted than potassium to the manufacture of aluminium, is exclusively employed for that purpose. Dumas states that the cost of making sodium need not exceed seven francs, per kilogramme, (which is equal to about two and one-fifth avoirdupois lbs.,) and that its manufacture is easier than that of phosphorus, and as simple as that of zinc.

Schroetter, in an essay upon the present state of the manufacture of aluminium in France, read before the Imperial Academy of Sciences of Vienna, states that the price at which sodium is manufactured by Deville is nine francs per kilogramme, and that three parts of this metal are required to make one of aluminium.

The Emperor of France with that liberality for science which distinguished Napoleon the First, by whose aid practical chemistry enjoys, even now, the soda, beet root, sugar, and other manufactures, placed in the hands of Deville, an unlimited credit for carrying out his experiments upon the manufacture of aluminium on a large scale. Let me state, for the benefit of army contractors, that Deville expended but thirty-six thousand francs of this credit. The condition of the manufacture in Paris in 1858 may be inferred from a collection of articles, made of aluminium, which were gathered for his government by the Austrian consul of that city. This collection comprised one hundred and fifty nine articles, at an aggregate value of seven thousand francs. It contained bars of the metal, cast and rolled cold; sheet, foil, and wire of great tenuity, and drawn tubes; spoons, forks, elegantly chased goblets, some of which were gilt, cups, bracelets, breastpins, studs, medallions, and a variety of other jewelry, in which the metal was pure or alloyed, and had been cast, stamped, drawn, filed, turned and engraved.

At this date there were in France two manufactories of aluminium; the original one under the direction of Paul Morin, founded by Deville, and situated near Paris; the other near Rouen, founded by William Martin, and carried on under the directien of the Brothers Tissier. The former establishment delivered sixty, the latter eighty kilogrammes of aluminium monthly. The price of the metal fell very rapidly from twelve hundred, to three hundred francs per kilogramme. Schroetter estimates that the price will sink to fifty francs, by the use of the mineral cryolite in the manufacture. Reckoning the franc at eighteen and three-fourth cents, this is equivalent to about three dollars and eighty cents per avoirdupois pound.

The methods originally proposed by Messieurs Deville and Morin, for the manufacture of aluminium, consisted in first forming the chloride of aluminium, which was placed in tubes, and decomposed there by the aid of sodium and heat, giving rise to chloride of sodium, or common salt, and aluminium. Soon after the value of the manufacture was ascertained, many improvements, some of which were patented, were introduced respecting materials, apparatus, and furnaces. The present processes in use may be reduced to two, which do not differ in principle from the original one. Alumina prepared from kaolin, or common clay, or these substances themselves, or finally alumina from any source, is mixed with charcoal and common salt, and is acted on in a heated state by chlorine gas. A double chloride of aluminium and sodium is thus formed. It is liquid and volatile by heat solidifying on cooling. By renewing the alumina mixtures in the retorts, the process may be carried on continuously like the gas manufacture. The resulting cake of double chloride is broken up, and mingled with the proper proportion of sodium, and is exposed to heat upon the sole of a reverberatory furnace, somewhat similar to that used in the manufacture of soda ash. The sodium abstracts the chlorine from the chloride of aluminium, forming common salt, with which is mixed the aluminium, found after cooling, in plates, globules, and powder. The separation of the metal takes place either mechanically, or by dissolving out the salt by water.

By this process *clay* is the crude ore of aluminium.

It was not long however before Rose, the distinguished chemist of Berlin, called attention to the mineral cryolite as the ore *par excellence*, of aluminium. This mineral is a double fluoride of aluminium and sodium, ($3\,NaFl, Al_2\,Fl_3$). When cryolite is heated with sodium, exactly the same reaction takes place as in the case of the double chloride of aluminium and sodium, but with the different result that fluoride of sodium is formed in place of common salt. By the use of this mineral there is, therefore, a saving of time, labor, and consequently of money in preparing the aluminium "*ore*."

Cryolite takes its name from the fact that it melts in the flame of a candle; the name means "ice-stone." It is found in large quantities on the coast ef Greenland, and has been imported into England by the soap manufacturers who make an alkaline lye from it. The owner of the mine has made a twenty years lease, to deliver three thousand tons of the mineral yearly in a French port, at three francs per hundred kilogrammes.* The fluoride of sodium, resulting from the manufacture of aluminium from cryolite, is a valuable product, as it may be transformed into *soda* and *fluor spar.*

The properties of aluminium make it extremely valuable for dental purposes. It resists sulphur emanations, and all means which promote the oxidation of most metals. It is soluble with great difficulty in all acids except hydrochloric. Caustic potassa attacks it; but it is not oftener exposed to this reagent than platinum, which behaves in a somewhat similar manner. It is persistent in the air. Werther proved this by the use of a weight, made from fine aluminium wire, which in five years constant use with his chemical balance did not exhibit any alteration of weight.

It may be hammered into a thin leaf, or foil of any required thickness, rolled, drawn into wire, filed, cast &c., subjecting it to whatever gold can bear.

The conducting power for heat of silver being one thousand, that of aluminium is six hundred and sixty five, which is less than that of gold, though greater than that of platina. Its density is one fourth that of silver. This is one of its most valuable properties for use in the plates of artificial teeth. Articles made of this metal are surprisingly "light." It is a metal of the lowest specific gravity of any employed in the arts. Its tenacity is great. Its products of oxydation are not poisonous, like those of copper and some other metals. By oxydation it becomes converted into alumina, which is a tasteless, inodorous compound, insoluble in water and ammonia; soluble in strong acids and in caustic potassa.,

The alloys of aluminium are very valuable. That made of three parts of the metal to one hundred of tin is harder than tin, and resists better the action of acids.

Five parts of silver with one hundred of aluminium is hard and elastic. It has been used for fruit and dessert

*Jour. Pr'o Pf. Ch. 73 p 501.

knives. An alloy of one hundred silver and five aluminium is peculiarly adapted to coinage. It is expected that aluminium will eventually supercede copper in coins, owing to the beauty, durability, and other advantages of the alloy.

The most valuable alloy of this metal is *aluminium bronze;* a compound of copper and aluminium. With from five to ten per cent. of aluminium, the alloy closely resembles gold in color; and is remarkable for its hardness, tenacity, elasticity, and unchangeability in the air, in acids and in solutions of salts. It may be forged when hot, and possesses a hardness and tenacity little inferior to that of steel. Pistol and rifle barrels have been made from it. This alloy has been used for parts of astronomical instruments which require scales to be engraved upon them. There are other alloys of aluminium, less important than the foregoing, which must be passed by for want of time.

If aluminium be sold at one hundred francs per kilogramme, silver costs two and one-fifth times as much; but owing to the lower density of the former metal, aluminium is practically one-ninth the value of silver, taking the above mentioned ratio of cost.

The little time allowed for writing this article has prevented an investigation into the state of the aluminium manufacture in this country. I have learned that a couple of years ago a manufactory of the metal was commenced in Camden opposite Philadelphia, but I know not what success attended it.

I have no doubt that the introduction of aluminium to the arts, will be accepted hereafter as one of the most valuable scientific contributions of the first half of the present century, which has been so fruitful in inventions useful to mankind.

2. The material for filling teeth has been improved in the substitition of spongy for leaf gold. This material is well known, a patent having been taken for it in this country, and improvements upon its manufacture having been published in the English scientific journals.

With the exception of the amalgams, which do not always give satisfaction, it is the only improvement made in late years upon the material for filling teeth, and yet the present state of chemical science is most favorable to the discovery of a still greater improvement.

For this purpose there is needed a white and not porous substance, soft enough to be applied to the cavity, becoming rapidly hard, and not acted upon by the fluids of the mouth. It must adhere closely to the tooth, must contain nothing poisonous, nor yield any such compound, and must be a poor conductor of heat.

The following cement has been proposed for this purpose by M. Feichtinger (Repertoire de Pharmacie.) Make a well mingled impalpable powder containing glass one part, oxide of zinc (free from carbonate) three parts. Make also a solution of chloride of zinc (density 1-5 to 1.6) fifty parts, borax one part. In preparing the liquid, dsisolve the borax in a little hot water and add it to the chloride of zinc. The borate of zinc is at first precipitated, but redissolves by shaking. To make the cement, add enough of this liquid to the powder to make a stiff paste. As it quickly hardens, too much of the cement must not be mixed at one time. If the ingredients are pure the cement is perfectly white, becomes after a short time as hard as marble, and remains so even after prolonged contact with water.

If the material for filling carious teeth is susceptible of improvement, what shall be said of the knowledge of the nature of the cause, and of the means of preventing and arresting the decay? Let those who are more capable than the writer answer the question.

Nevertheless, chemists have given hints for investigating the subject.

The universal belief prevails, that caries is a disease; the very derivation of the name itself suggests this hypothesis. I have often been impressed, when examining Morton's extensive collection of crani in the Academy of Natural Sciences in Philadelphia, with the excellence of the teeth of the ancient, and of the uncivilized specimens. Has modern civilization, by introducing a new diet, and new modes of life, rendered the dentine more susceptible to disease; or have these influences brought the teeth in contact with corrosive substances not formerly present? May not both causes combine to hasten this decay? The dentine is of different degrees of hardness with different individuals. This quality is doubtless hereditary. A certain diet and mode of life may favor the formation of a less compact dentine, and this quality may descend to posterity, and become intensified and confirmed by the transmission. A dentine possessing a weaker resistance to comminution may be more susceptible to corrosive influences, as it may also be to disintegration by disease. Statistics would doubtless throw much light upon this vexed question.

Baron von Reichenbach, a distinguished chemist, has, in his declining years, directed a wish to chemists, which will be found on the two hundred and forty ninth page of the seventy seventh volume of the Journal der Practische Chemie. It has so peculiar an interest attached to it, whatever may be thought of the value of its suggestions, that I may be pardoned in translating the, greater part of it, giving as much as is consistent with proper brevity, the Baron's own words.

"Many of our distinguished chemists present us daily with such excellent investigations, upon subjects so remote from our necessities displaying thereby so much industry and acumen, that it often happens that one must deplore that so much talent passes by subjects lying much nearer to us, and of which the accurate investigation frequently belongs to the most pressing needs of mankind. * * * Such a subject is the detested enemy which attacks our teeth, springing from well concealed ambushes and laying waste the power of nutrition, as well as corporeal beauty. I allude to the so-called *caries of the teeth.* Few persons are so happy as to be free from it; and all are acquainted with this sorrow, which reaches the youngest as well as the oldest; which disfigures the noblest countenances; which renders mastication difficult or even impossible, and which defiles the breath. This affection, which still resists all the endeavors of the physician, has been placed within the domain of pathology, and is regarded as a disease of organic nature, being treated as a kind of bone ulceration or gangrene. It is even maintained that there are several varieties of the disease, but notwithstanding, therapeutics have not in the slightest prevailed against them. The disease has been regarded as a suffering derived from the general state of the constitution, and physicians have spoken of diet, fresh air, of baths and journeys, as good for its alleviation. Fifty years ago, I was acquainted with a blooming maiden, abounding in health and vital activity, whose two front teeth began to decay upon a small point of contact. The tooth powder of Hufeland, which contains Peruvian bark, was used industriously; but the evil constantly increased, and the young lady was very anxious and disturbed at the prospective loss of her front teeth. I recommended the use of charcoal powder, which at once arrested the decay. The lady died, in her forty-sixth year, after a long illness, and shortly before her death I saw the teeth in the same condition in which they had been left by the arrest of the caries in her eighteenth year. Charcoal powder seems, then, to be able to arrest caries; but it can only avail in favorable conditions of application, which are confined to the front teeth, and it is is not always available even to these. To strike at the foundation of the evil, it is of imperative necessity to learn its nature. The dentists tell us but little of it, for they have fathomed it but little.

An instance accurred sometime ago in Vienna, which throws a ray of light into this darkness. An actress, to replace a loss so disastrous to one of her profession, procured a set of artificial teeth made of walrus tusk. After using them for a few years, she complained to her dentist that the artificial teeth were beginning to be affected with decay. Incredible as it may appear, two teeth in contact were found to be affected with caries, exactly as in the case of living teeth! I have seen this set of teeth myself; Dr. Heider, a dentist of Vienna, preserves them as a curiosity. This example is of great value; for it teaches that caries is independent of the living organism, and can not be a subject of pathology, but is one of chemistry, and that its source must be sought, not in vital activity, but in chemistry. In the meantime two score years have elapsed, and dentistry has made no use of this interesting discovery. The fact remains to-day as a curiosity, and no one has taken it as a point of departure for further investigation.

Not long ago I embraced the opportunity of the loss of some back teeth to examine their fragments. I found that the substance coating the cavities reacted acid to litmus; the deeper I penetrated the substance of the tooth the more acid was the reaction. At length the coating was entirely removed, and the bare, corroded tooth cavity displayed the acid reaction the strongest. From this it seemed that it was either an organic acid which dissolved the dentine immediately, or that the decay generated an acid; or at all events that the decay was developed under the influence of acid activity. The peculiar whitish coating with which the cavity was clothed can not be a result of the food, for it bore no characteristics of putrefaction, but appeared to be a part of the tooth, or a residue of its solution, and was throughout penetrated by, or exclusively saturated with acid.

In the seat of the affection, therefore, there resides an acid inimical to the chemical composition of the tooth. What kind of an acid is it; what are its properties; whence does it come, and how does it reach the inside of the tooth; how does it effect its injury?

First let us determine with what the tooth comes chiefly in contact. It is with air, with the carbonic acid of the breath, with saliva and food. Air and carbonic acid can not injure it ; it has been organized with a view to these gasses. The same is true of saliva so far as it is of a normal character. But the food is of varied nature. It would have been interesting to have questioned the Viennese actress closely as to her mode of life and diet. The food which we enjoy is almost always more or less acid. All vegetables and vegetable matter, fru ts, drinks, even ordinary water are somewhat acid, and if meats are not so we eat with them so much salad, herbs, roots, and vegetable juices, that they become essentially as acid as the rest of our food. We also inhale carbonic acid with our breath, even if we except that which we exhale by the action of the acidifying oxygen upon the elements of the blood. In short, what constantly reaches our teeth is acid; alkaline food is never taken, for if there be alkaloids in some ef our spices they are found there neutralized by their respective acids.

This much therefore is true, that our teeth remain under the constant influence of acids, and are, so to say, in a constant bath of acidulous juices. If then an acid is the means of the destruction of our teeth, it obtains full support through the whole course of our life, and never do we take in the mouth anything counteracting this acid activity (?) I know of but one instance where this was done, viz : in the case of the lady to whom I recommended the use of charcoal tooth powder. Hufeland's powder contains in its substance vegetable acids, but charcoal contains carbonate of potassa, and is in a condition to neutralize the acids which redden litmus. It is therefore probable that the maiden, by its employment, destroyed the acid decaying her teeth by neutralizing it. This was possible from the position of the decay, which was in front and readily accessible to the tooth brush. In cases where the decay has attacked a molar, or where it is between the teeth, or has already excavated a deep cavity, neither charcoal powder nor the tooth brush can reach it, nor can the small amount of carbonate of potassa contained in the charcoal act well upon the acid, especially through the deposit clothing the cavity. Soap has been recommended as a tooth wash; if it is efficient, the acid in the tooth must have a stronger affinity for potassa than fatty acids in soap, which would, in such a case, be more efficient than charcoal powder.

By following the course of decay, it is apparent, that caries can not be putrefaction, because nothing ammoniacal is evolved; the affected spot has no bad odor, for the impure breath of persons with decayed teeth arises, not from the decay, but from the remains of food adhering to the cavities, and there decomposing.

The progress of the evil can not be determined by the immediate contact of the food; because this contact is soon prevented by the deposit which covers the walls of the cavities. It is only liquids which can penetrate the deposit and reach the substance of the tooth. If the destroying acid proceeds directly from the food, we must, by chemical laws, assume that it neutralizes itself upon the wound, combining with the substance of the tooth. Since the evil progresses uninteruptedly, we must assume that the acid is constantly presented to the wound by mastication, which presses the neutralized liquids from the cavities, filling them with fresh acid. * * * Filling the cavities with gold arrests the progress of the decay by preventing the contact of acid.

It is a question for investigation whether the injurious acid be presented to the teeth ready made; or whether it be formed upon the seat of the decay by decomposition. At all events, the teeth can not universally offer points of attack to it, else the decay would be general, and diffused through all the teeth. There must be peculiar conditions favorable to the action of the acid in certain parts of the teeth.

In the investigation of this subject it might be well to examine the conditions under which caries attacks the teeth of our domestic animals; to examine their food, and under what conditions it is taken. There must be veterinary experience upon this subject. Farmers declare that sheep lose their teeth when long fed upon hot mashes. I have long used a solution of carbonate of soda sharpened with a little caustic potassa, applying it with a tooth brush after each meal, with the expectation of neutralizing whatever acid might remain in the mouth after eating. I soon fell upon a better way of applying the alkali, viz: by gently chewing a piece of sponge saturated with it. It was to be expected that by this means the acid would be more perfectly neutralized through the deposit coating the cavities of the teeth. I have lost no teeth since adopting this practice, and time will show whether it be efficient.

It follows from all that we can not doubt the possibility of relieving the human family of a great amount of suffering arising from carious teeth, and that the case of the Viennese actress indicates the prospect of success; that the evil is not constitutional, but chemical; that it is a phenomenon of acid characters, and that it is within the immediate province of chemistry to throw light upon the subject."

Reichenbach concludes with an earnest appeal to chemists to take up the subject, saying, that there can be no lack of substance upon which to operate for the examination of the acids when so many teeth are extracted annually by the dentists.

It may be remarked as a novelty that the Baron attributes the effciency of charcoal powder to its alkali, and not, as generally assumed, to its anti-septic property.

He does not allude to the solvent power of sugar upon the inorganic salts forming the basis of the teeth.

It is strange that he (especially in the character of a German) should assert that alkaline substances never reach the teeth. Every tobacco smoker knows to the contrary, and may demonstrate it while smoking by the instant bluing of a piece of red litmus paper placed upon the tongue. Leaving out of consideration the excessive use of soda in baking, there is another source of alkali in the mouth, viz: the saliva, which is constantly more or less alkaline, except when in fasting it becomes neutral and even sometimes acid. The alkalinity of the saliva increases during eating and immediately afterward.* Frerichs collected one hundred grammes of saliva while smoking, and found that it required fifteen centigrammes of sulphuric acid to neutralize the alkali which it contained.

It is possible that the alkaline nature of saliva, especially after eating, was intended by the Creator to neutralize acids destructive to our teeth (which are composed chiefly of carbonate and phosphate of lime) which would arise from the food, and that the circumstances of civilized life have destroyed the balance existing between the acidity of the food, and the alkalinity of the saliva. Let it not be objected that in such a case Providence has shown its aversion to civilization, for brains have been given to us to use for obviating the difficulty by discovering means for the preservation of the teeth.

If an alkali favors this preservation, the reputed freedom of the teeth of habitual smokers from serious decay, may be accounted for. As it is customary to use this luxury especially after eating, the alkali is thus presented when most needed. I attribute the preservation of my own teeth to smoking. When twenty two years of age I had never used tobacco in any form ; for a period of fifteen years I have not passed five days without smoking. My teeth at twenty two were nearly all affected with caries.

Dr. Neill of Philadelphia, who attended to them, filling every cavity with gold, informed me that the dentine was extremely hard and compact, and that I would retain the use of my teeth for a long time. I have never lost but one of the fillings which he placed, some of them in difficult positions, and I have often attributed my fortune in this respect to the good habit of smoking, a habit commenced on purely philosophical ground, and which has never been repented of.

---

* See many authorities in Gmelin.

Reichenbach's appeal for the chemical investigation of caries can not be urged upon the dental profession. But nevertheless, the five thousand dentists of our country can aid in the investigation, and even determine the course which it shall pursue, if they will collect statistical information about caries.* It would not occupy much time for each practicing dentist to note down in a book, ruled for the purpose, answers to certain questions proposed to their patients respecting diet, mode of life, business, the time at which decay commenced, its rapidity, whether it be hereditary &c., &c.

Such information collected by the dentists, and generalized, and simplified by the action of their Societies and Conventions, can not fail to result in benefit to mankind.

